# Biological Systems of Vitamin K: A Plasma Nutriproteomics Study of Subclinical Vitamin K Deficiency in 500 Nepalese Children

**DOI:** 10.1089/omi.2015.0178

**Published:** 2016-04-01

**Authors:** Sun Eun Lee, Kerry J. Schulze, Robert N. Cole, Lee S. F. Wu, James D. Yager, John Groopman, Parul Christian, Keith P. West

**Affiliations:** ^1^ Center for Human Nutrition, Departments of International Health, Johns Hopkins Bloomberg School of Public Health, Baltimore, Maryland.; ^2^ Mass Spectrometry and Proteomics Core Facility, Department of Biological Chemistry, Johns Hopkins School of Medicine, Baltimore, Maryland.; ^3^ Environmental Health Sciences, Johns Hopkins Bloomberg School of Public Health, Baltimore, Maryland.

## Abstract

Vitamin K (VK) is a fat-soluble vitamin whose deficiency disrupts coagulation and may disturb bone and cardiovascular health. However, the scale and systems affected by VK deficiency in pediatric populations remains unclear. We conducted a study of the plasma proteome of 500 Nepalese children 6–8 years of age (male/female ratio = 0.99) to identify proteins associated with VK status. We measured the concentrations of plasma lipids and protein induced by VK absence-II (PIVKA-II) and correlated relative abundance of proteins quantified by mass spectrometry with PIVKA-II. VK deficiency (PIVKA-II >2 μg/L) was associated with a higher abundance of low-density lipoproteins, total cholesterol, and triglyceride concentrations (*p* < 0.01). Among 978 proteins observed in >10% of the children, five proteins were associated with PIVKA-II and seven proteins were differentially abundant between VK deficient versus sufficient children, including coagulation factor-II, hemoglobin, and vascular endothelial cadherin, passing a false discovery rate (FDR) threshold of 10% (q < 0.10). Among 27 proteins associated with PIVKA-II or VK deficiency at a less stringent FDR (q < 0.20), a network comprised of hemoglobin subunits and erythrocyte anti-oxidative enzymes were highly and positively correlated each other (all r > 0.7). Untargeted proteomics offers a novel systems approach to elucidating biological processes of coagulation, vascularization, and erythrocyte oxidative stress related to VK status. The results may help elucidate subclinical metabolic disturbances related to VK deficiency in populations.

## Introduction

Vitamin K (VK) is a fat-soluble micronutrient, known primarily for its function in blood coagulation (FAO/WHO, 2002). Relatively little public health attention has been paid to vitamin K status and its health effects, mainly because hemorrhaging, a clinical manifestation of vitamin K deficiency (VKD), is rare in healthy populations (Suttie, 2009). However, there is evidence that young infants have a risk of VKD bleeding, especially among exclusively breast-fed infants not receiving VK prophylaxis at birth in disadvantaged populations (Shearer, 2009).

A growing number of studies have shown that low vitamin K intake or plasma concentration is associated with increased risk of aging-related health outcomes including cardiovascular diseases, hip fractures, insulin resistance, inflammation, and cognitive impairment in adult and elderly populations (Booth, 2009; Ferland, 2012a, 2012b; McCann and Ames, 2009). In addition, a sufficient number of studies have reported an association of VK status with markers of bone metabolism and bone mineral content, suggesting a role in bone health in growing children (Cashman, 2005; Kalkwarf et al., 2004; O'Connor et al., 2007). These findings collectively suggest that suboptimal VK status may be a public health problem that affects diverse health outcomes across the life stages beyond its classical functions in coagulation.

Vitamin K exerts its functions through a unique mechanism. Different from other fat-soluble vitamins, it serves as a cofactor of ϒ-glutamyl carboxylase that converts glutamic acid (Glu) residues into ϒ-carboxyglutamate (Gla) residues, allowing calcium-dependent conformational changes of VK-dependent proteins (VKDPs) (Stenflo et al., 1974; Suttie, 1980). The Gla residues have a profound role in the biological activity of VKDPs, as this post-translational modification promotes VKDP localization and binding to membranes where they exert their functions (Presnell and Stafford, 2002).

Of at least 15 VKDPs identified, 7 proteins are either blood coagulants (factor II, X, VII, and IX) or anti-coagulants (Protein C, S, and Z), all carboxylated in the liver (McCann and Ames, 2009). Some extrahepatic VKDPs are extracellular matrix proteins functioning as regulators of bone mineral maturation and inhibitors of calcification of smooth muscle or vascular endothelium, but the physiological functions of others remain elusive (Ferland, 2012a; McCann and Ames, 2009).

Among hepatic VKDPs, prothrombin (factor II) is the most well-characterized and abundant in circulation (Suttie, 2009). When the liver storage of VK is depleted, it secretes under-carboxylated prothrombin into plasma, which is called “protein induced by vitamin K absence-II” (PIVKA-II) (Hemker et al., 1970). Because prothrombin has 10 Gla residues, PIVKA-II can be in different forms, and only a few un-carboxylated glutamate residues can substantially reduce the coagulant activity of prothrombin (Esnouf and Prowse, 1977). This biologically nonfunctional form of prothrombin can be detected before the physiological function of the pro-coagulant pathway is disrupted, suggesting more sensitivity to subclinical VK status than prothrombin time, which is used to test blood clotting time (Suttie, 1992). Thus, plasma PIVKA-II has been used as an indicator of suboptimal vitamin K status (Dituri et al., 2012).

The plasma proteome may provide a unique opportunity to explore biological roles of vitamin K in populations. It is the most comprehensive proteome among human body fluid proteomes, containing not only classic proteins synthesized by the liver and secreted into the circulation, but also proteins leaked from many different types of extrahepatic tissues (Anderson and Anderson, 2002). We have previously shown that plasma proteomics reliably discerns proteins known to circulate in association with vitamin A, E, and D, selenium and copper and may uncover nutrient-associated biological pathways that to date have not been appreciated (Cole et al., 2013; Schulze et al., 2015; West et al., 2015). Although there is no known plasma carrier protein for vitamin K (Olson, 1984), a plasma proteome may reflect changes in VKDPs or homeostatic changes in the local tissue environment in response to suboptimal functions of VKDPs.

In this study, we attempted to identify proteins associated with subclinical VKD assessed by elevated plasma PIVKA-II. We previously reported that subclinical VKD exists in approximately 20% of undernourished, but otherwise relatively healthy, children living in rural Nepal where multiple micronutrient deficiencies are common (Schulze et al., 2014). The results of this study may help elucidate the biological processes and potential health consequences related to suboptimal vitamin K status in healthy populations.

## Methods

### Study design, participation, and sample collection

In 1999–2001, we carried out a community-based, cluster-randomized controlled trial of antenatal micronutrient supplementation to improve birth size and to reduce risk of low birth weight in the southern plains district of rural Sarlahi, Nepal (ClinicalTrials.gov:NCT0011527) (Christian et al., 2003). In 2006–2008, 3524 children 6–8 years of age, born to mothers who participated in the trial were followed-up for growth, nutrition, and health assessment. Details of the follow-up have been described elsewhere (Stewart et al., 2009a; Stewart et al., 2009b).

The following information was collected by field workers during home visits: ethnicity, household socioeconomic characteristics, child 7-day morbidity (number of days with particular symptoms), literacy, and past week dietary intake (frequency of consumption of key foods). Trained anthropometrists measured height, weight, and mid-upper arm circumference using previously reported methods (Stewart et al., 2009a). Early morning venous blood samples (10 mL in sodium heparin-containing tubes without preservatives or antioxidants) were collected by phlebotomists, brought to a field laboratory and centrifuged. Blood plasma was aliquoted, immediately frozen under liquid nitrogen, shipped to the Johns Hopkins University Center for Human Nutrition, and stored at −80°C for future laboratory analysis.

Details of specimen selection for proteomics assessment protocol have been described (Cole et al., 2013; West et al., 2015). Of the 3305 children whose blood samples were obtained, 2130 children had the full four aliquots of plasma specimens, birth weight measured within 72 hours after birth, and complete epidemiological data from both the original trial and follow-up study. Among them, 1000 children were randomly sampled for micronutrient assessment. Finally, 500 of those children—balanced across maternal intervention groups—were randomly selected for the proteomics analysis.

### Ethics, consent and permissions

Study field staff explained the research purpose, activities and risks of participation to mothers of eligible children and obtained oral informed consent during the child follow-up household visits (Stewart et al., 2009a; 2009b). The follow-up study including biospecimen collection and laboratory assessments received ethical approval from the institutional review board (IRB) at Johns Hopkins University, Baltimore, MD, USA and the Nepal Health Research Council (NHRC) in Kathmandu, Nepal.

### Assessment of protein induced by Vitamin K absence-II (PIVKA-II), lipids, and inflammatory markers

PIVKA-II in plasma samples was quantified using an adaptation of a commercial enzyme immunoassay (i.e., more low concentration standards were added to improve resolution at low concentrations) (ASSERACHROM, Diagnostica Stago, France). The inter-assay coefficient of variability (CV) was 15.0% for a quality control pool at 3.3 μg/L, designed to be close to the cutoff for VKD. PIVKA-II concentration values less than 0.001 μg/L were undetectable (*n* = 26), leaving 474 plasma samples available for analysis with a continuous outcome. Details of lipid profiles (total cholesterol, high-density and low-density lipoprotein cholesterol, and triglycerides) and inflammation markers (C-reactive protein and α-1-acid glycoprotein) assessment have been reported elsewhere (Schulze et al., 2014; Stewart et al., 2009b).

### Plasma proteomics assays

Quantitative proteomic analysis of these plasma samples has been reported in detail elsewhere (Cole et al., 2013; Lee et al., 2015; West et al., 2015). Briefly, plasma samples from each of the 500 participants randomly chosen for proteomics evaluation were immunoaffinity-depleted of six high abundance proteins using a Multiple Affinity Removal System LC column-Human 6 (Agilent Technologies). Depleted protein extracts went through overnight trypsin digestion and were labeled in random order with isobaric tag for relative and absolute quantitation (iTRAQ) 8-plex reagents (AB Sciex).

The samples were mixed and fractionated by strong cation exchange chromatography and analyzed on a LTQ Orbitrap Velos mass spectrometer (MS) (ThermoScientific, www.thermo.com/orbitrap). MS/MS spectra were extracted using Thermo Scientific Xtract software. All spectra data was searched with Proteome Discoverer software (v1.3, Thermo Scientific) against the RefSeq database using the Mascot search algorithm (Matrix Science, v2.1). Data were obtained from 72 iTRAQ experiments and the average number of proteins per experiment was 589. A total of 4705 proteins were detected with varying missing values.

### Statistical analysis

Estimation of relative abundance of proteins has been documented in detail elsewhere (Cole et al., 2013; Herbrich et al., 2013). Briefly, log 2 base transformed-reporter ion intensities were median normalized in a single iTRAQ experiment. We applied linear mixed effects models (LME) to combine the proteomics data from different experiments and to estimate the association of protein relative abundances with plasma PIVKA-II concentration. Because the distribution of PIVKA-II data was skewed to the right, we used log 2 base transformed plasma PIVKA-II data in the analysis. We fitted univariate random intercept models with PIVKA-II as a dependent variable and protein as an independent variable. Parameters were estimated using restricted maximum likelihood estimation.

From the LME model, the fixed effect of the slope of the PIVKA-II and protein association was summarized in tables and interpreted as percentage change in PIVKA-II associated with doubling of relative abundance of protein. The strength of association was denoted by *p* value. A false discovery rate (FDR)-corrected *p* value was computed and denoted as q in tables (Stroey, 2002). We considered proteins with q < 0.10 to be significantly associated with PIVKA-II or VKD. R^2^ refers to the proportion of variance explained by the fitted values of the nutrient:protein regression models (Robinson, 1991).

In addition, we categorized children into VKD (PIVKA-II >2 μg/L) and sufficient groups (PIVKA-II ≤2 μg/L) to identify proteins that may not be linearly associated with PIVKA-II concentration, but differentially abundant by VK status (Schulze et al., 2014). We built a correlation matrix with proteins associated with PIVKA-II or VKD passing a relaxed threshold of q less than 0.20 to explore potential biological pathways or metabolic networks among proteins associated with VK status. Correlation coefficients of pairwise protein:protein were calculated within each iTRAQ experiment and the averaged coefficients across iTRAQ experiments were used to construct a correlation matrix. The dataset of plasma PIVKA-II concentrations and relative abundance of proteins is available in Supplementary Table S1 (supplementary material is available online at www.liebertpub.com/omi). All analyses were carried out using in-house-developed open source software implemented in the statistical environment R (R Development Core Team, 2013).

## Results

The median (interquartile range) of plasma PIVKA-II concentration was 1.31 (0.83, 1.87), ranging from 0 to 13.0 μg/L. Demographic, anthropometric, household, dietary, and health characteristics and lipid and inflammatory profiles of children are compared by vitamin K status of children (PIVKA-II > or ≤2 μg/L) in Table 1. There were no significant differences in most characteristics including gender and ethnicity between the two groups, except that children with VKD were slightly younger (*p* = 0.001) and reported a higher prevalence of fever in the previous week (*p* = 0.010) than children with vitamin K sufficiency. For lipid profiles, low-density lipoprotein (LDL), total cholesterol, and triglyceride concentrations were significantly higher in children with VKD (all *p* < 0.01); high-density lipoprotein (HDL) cholesterol did not differ by groups (*p* = 0.588). Prevalence of elevated concentrations of inflammation biomarkers such as C-reactive protein and α-1-acid glycoprotein were not different by groups.

**Table 1.** Demographic, Anthropometric, Household, Dietary, and Health Characteristics of Nepalese Children by Vitamin K Status^a,b^

*Child characteristics*	*Deficient (*n* = 100)*	*Sufficient (*n* = 400)*	*P*^c^
*Demographic*			
Male, %	50.0	49.8	1.000
Age, years	7.3 (0.5)	7.5 (0.4)	0.001
*Anthropometric measurements*			
Weight, kg	18.3 (3.6)	18.3 (2.3)	0.912
Height, cm	113.8 (7.5)	114.2 (5.5)	0.640
BMI, kg/m^2^	14.1 (1.5)	14.0 (1.0)	0.498
MUAC, cm	15.4 (1.5)	15.5 (1.1)	0.768
*Undernutrition***^d^ %**			
Stunting (HAZ< −2)	37.0	39.5	0.731
Underweight (WAZ< −2)	44.0	49.5	0.383
Low BMI (BMIZ< −2)	15.0	16.8	0.786
*Education***%**			
Ever sent to school	62.0	68.0	0.462
Literacy	18.0	17.3	0.977
*Ethnicity of household* %			0.791
Pahadi	30.0	31.3	
Madheshi	70.0	68.7	
*Economic of household %*			
Electricity	50.0	51.3	0.911
Land ownership	71.0	78.5	0.144
*Diet, any intake in the past week %*			
Milk	70.0	68.3	0.828
Chicken	20.0	19.8	1.000
Fish	20.0	22.0	0.765
Other meat	31.0	32.5	0.867
Eggs	14.0	18.8	0.335
Dark green leafy vegetable	74.0	72.0	0.783
*Morbidity, any symptom reported in the past week %*			
Poor appetite	12.0	12.3	1.000
Fever	15.0	6.5	0.010
Diarrhea	2.0	3.5	0.657
Any symptom^e^	24.0	24.3	1.000
*Lipid profiles*^f^			
HDL cholesterol, mmol/L	0.71 (0.57, 0.88)	0.73 (0.60, 0.88)	0.588
LDL cholesterol, mmol/L	1.97 (1.79, 2.20)	1.84 (1.58, 2.10)	0.008
Total cholesterol, mmol/L	3.29 (2.94, 3.50)	3.10 (2.80, 3.37)	0.007
Triglyceride, mmol/L	1.13 (0.88, 1.58)	1.02 (0.78, 1.31)	0.006
*Infection/inflammation, %*			
CRP >5 mg/L	6.0	6.0	1.000
AGP >1 g/L	35.0	28.5	0.251

AGP, α-1-acid glycoprotein; BMI, body mass index; BMIZ, body mass index z score; CRP, C-reactive protein; HAZ, height-for-age z score; HDL, high-density lipoprotein; LDL, low-density lipoprotein; PIVKA-II, protein induced vitamin K absence-II; WAZ, weight-for-age z score.

^a^ Children were divided into vitamin K deficient (PIVKA-II >2 μg/L) and sufficient (PIVKA-II ≤2 μg/L) groups.

^b^ Data are expressed as mean (standard deviation) or median (interquartile range) unless otherwise indicated.

^c^ *P* value for group difference using t-test for continuous variables with normal distributions, Mann-Whitney test for continuous variables with skewed distributions, and chi-square test for categorical variables.

^d^ Anthropometric Z-scores were calculated based on the WHO reference for children 5–19 years of age.

^e^ Any symptom was defined as any symptom of poor appetite, vomiting, high fever, diarrhea, blood or white mucus in stool, productive cough, rapid breathing, blood in sputum, or painful urination in the past week.

^f^ Data are missing for HDL (*n* = 39), LDL (*n* = 176), total cholesterol (*n* = 149), and triglyceride (*n* = 28).

Among 978 plasma proteins identified and quantified in >10% of children (i.e. n > 50 children), five proteins were significantly associated with plasma concentration of PIVKA-II with at q < 0.10 (Table 2). Prothrombin or coagulation factor-II (F2) was most strongly associated with PIVKA-II, with a 137.5% increase in PIVKA-II concentration per 100% increase in relative abundance of F2. We also identified and quantified all seven hepatic pro- (coagulant factor II,VII, IX, and X) and anti-coagulation (protein C, S, and Z) VKDPs, but none were associated with PIVKA-II. Hemoglobin delta unit (HBD) was positively associated with PIVKA-II concentration with an 11.7% increase in PIVKA-II per 100% increase in relative abundance of HBD.

**Table 2.** Plasma Proteins Associated with Plasma PIVKA-II in Nepalese Children 6–8 Years of Age (q < 0.10)

*Protein (Gene symbol)^a^*	*Accession*	n^b^	*Percent change*^c^	*P*^d^	q^e^	*R*^2,f^	*Molecular/Biological function*
Prothrombin or coagulation factor II (F2)	4503635	474	137.5 (50.1, 275.8)	0.0002	0.0556	0.29	Blood coagulation
Cadherin 5, type 2 (vascular endothelium) (CDH5)	166362713	474	−39.8 (−54.6, −20.1)	0.0004	0.0649	0.29	Control intercellular junctions
Calcium channel, voltage-dependent, alpha 2/delta1 (CACNA2D1)	54112390	200	−46.0 (−61.7, −23.8)	0.0005	0.0649	0.30	Mediate Ca^2+^ influx
Gelsolin (GSN)	4504165	467	−38.6 (−53.8, −18.6)	0.0007	0.0895	0.30	Regulate and severe actin filaments
Hemoglobin, delta (HBD)	4504351	211	11.7 (4.7, 19.1)	0.0008	0.0921	0.27	Transport O_2_

Ca, calcium; O_2_, oxygen; PIVKA-II, protein induced vitamin K absence-II.

^a^ Putative prostaglandin E synthase 3-like (PTGES3; Protein GI accession number: 217416407) was also detected in 67 subjects (R^2^ = 0.36, *p* < 1 × 10^−5^, q < 0.01).

^b^ Maximum number of observation was 474 (n = 26 zero values were dropped due to log2 transformation of plasma PIVKA-II).

^c^ Percent change in PIVKA-II concentration with doubling (100% increase) of relative abundance of protein.

^d^ *P* value for hypothesis testing of a null association between PIVKA-II concentration and relative abundance of protein using a linear mixed model.

^e^ Multiple hypothesis testing was corrected using false discovery rate.

^f^ Variance in PIVKA-II concentration explained by protein.

For negative correlates, a 40%–45% decrease in PIVKA-II was associated with a 100% increase in relative abundance of vascular endothelium cadherin 5 (CDH5), voltage-dependent calcium channel α2δ1 (CACNA2D1), and gelsolin (GSN). Individually, these proteins each explained about 30% of variability in PIVKA-II concentration in plasma. Another fifteen proteins were positively and negatively associated with PIVKA-II when a relaxed discovery threshold (q < 0.20) was applied, including α-1-B glycoprotein (A1BG), another hemoglobin subunit α1 (HBA1), endogenous antioxidative enzymes (catalase [CAT] and peroxiredoxin 2 [PRDX2]), carbonic anhydrase 2 (CA2), and cytoplasm/intracellular organelle proteins (Supplementary Table S2).

Seven proteins were differentially abundant by vitamin K status of children at q < 0.10 (Table 3). Endoplasmic reticulum protein 44, F2, inhibin beta E, zinc finger protein 645, heparin cofactor, and A1BG were approximately 4% ∼30% more abundant and CDH5 was 6% less abundant in the plasma of children with elevated PIVKA-II (>2 μg/L) relative to children with normal PIVKA-II concentration (≤2 μg/L). Another five proteins were differentially abundant by vitamin K status under a relaxed discovery threshold of q < 0.20, summarized in Supplementary Table S3.

**Table 3.** Differentially Abundant Plasma Proteins Between Children with Vitamin K Deficiency and Sufficiency (q < 0.10)^a^

*Protein (Gene symbol)*	*Accession*	n^b^	*Percent difference*^c^	*P*^d^	q^e^	*Molecular/Biological function*
Endoplasmic reticulum protein 44 (ERP44)	52487191	97	28.0 (14.2, 43.5)	2.16 × 10^−5^	0.0228	Control oxidative protein folding
Cadherin 5, type 2 (vascular endothelium) (CDH5)	166362713	500	−6.2 (−9.0, −3.4)	2.86 × 10^−5^	0.0228	Control intercellular junctions
Coagulation factor II (F2)	4503635	500	3.6 (1.7, 5.5)	0.0002	0.0612	Blood coagulation
Inhibin, beta E (INHBE)	13899338	97	30.4 (13.4, 50.0)	0.0002	0.0612	Regulate pituitary reproductive hormone secretion
Zinc finger protein 645 (ZNF645)	22749189	388	10.4 (4.7,16.4)	0.0003	0.0671	Testis-specific E3 ubiquitin-protein ligase
Heparin cofactor (SERPIND1)	73858566	500	5.6 (2.5,8.8)	0.0003	0.0671	Thrombin inhibitor
Alpha-1-B glycoprotein (A1BG)	21071030	500	3.6 (1.6, 5.6)	0.0004	0.0758	Unknown

PIVKA-II, protein induced vitamin K absence-II.

^a^ Vitamin K deficiency and sufficiency were defined as PIVKA-II concentration > and ≤2 μg/L, respectively.

^b^ Maximum number of observation was 500.

^c^ Percent difference in protein abundance in children with vitamin K deficiency relative to children with vitamin K sufficiency.

^d^ *P* value for hypothesis testing of no difference in relative protein abundance between two groups.

^e^ Multiple hypothesis testing was corrected using false discovery rate.

**FIG. 1.**
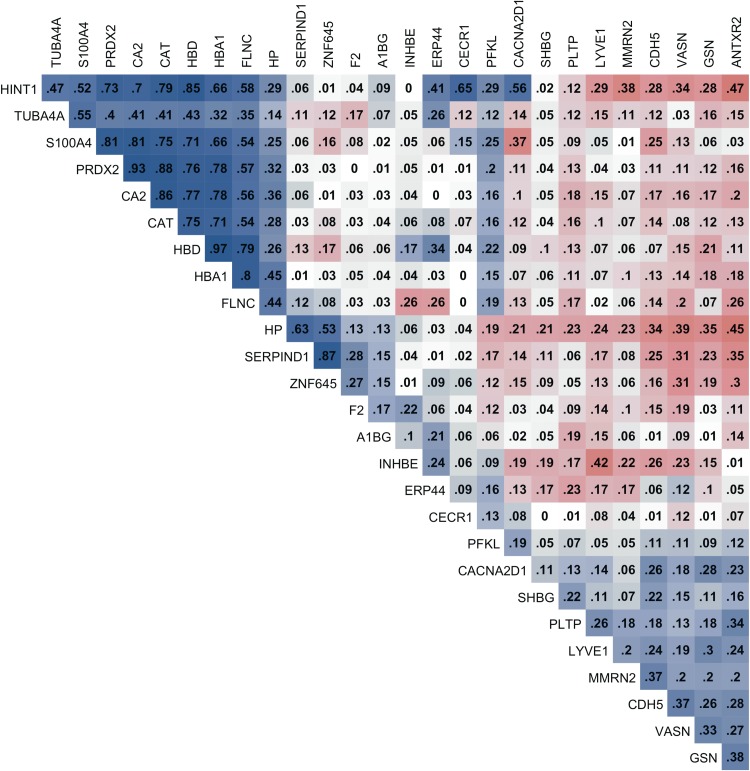
Correlation matrix of relative abundance of plasma proteins associated with vitamin K status. Plasma proteins were associated with plasma protein induced vitamin K absence-II (PIVKA-II) concentration or differentially abundant by vitamin K status (PIVKA-II> or ≤2 μg/L), passing a relaxed discovery threshold, q < 0.20 (*n* = 27). *Blue and red colors* depict positive and negative correlation coefficients, respectively, and *darker colors* represent stronger correlation. Correlation coefficients less than ±0.01 were denoted as 0.

The correlation matrix of proteins associated with PIVKA-II or VKD (PIVKA-II >2 μg/L) showed that there are prominent, strong positive correlations among hemoglobin subunits and endogenous enzymes such as HBD, HBA1, CAT, CA2, and PRDX2 (all correlation coefficients >0.7) (Fig. 1).

## Discussion

Vitamin K status in children has not been frequently discussed as a public health issue except regarding its role in bone health and prevention of infant bleeding disorders. This study explored the plasma proteome in children living in rural Nepal in association with subclinical VKD, assessed by plasma concentration of PIVKA-II, an indicator of hepatic vitamin K depletion. Because our measurement of VK status was indirect, and because VK has no known carrier proteins, we did not expect to find a strong VK proteome, unlike the case for other nutrients we have explored (Cole et al., 2013; Schulze et al., 2015; West et al., 2015).

Nonetheless, five proteins moderately predicted plasma concentration of PIVKA-II at a threshold that limited the chance of a false positive to below 10% (q < 0.10). One was a hepatic blood coagulation protein, validating the proteomics approach, while others were extrahepatic in origin, including erythrocytes, suggesting there may be a spectrum of potential biological responses to subclinical vitamin K deficiency.

Few population estimates of vitamin K status exist in low-income countries. In this rural setting of Nepal, 20% of early school-aged children were mildly vitamin K-deficient (PIVKA-II >2 μg/L), a prevalence that is comparable in magnitude to other co-existing micronutrient deficiencies in this population (Schulze et al., 2014), but exhibiting a far narrower and lower range of abnormal PIVKA-II values (i.e., 99% of PIVKA-II values ranged from 0 to 4 μg/L) than observed in children with hepato-billiary or inflammatory bowel diseases (Dougherty et al., 2010; Mager et al., 2006; Nowak et al., 2014; Strople et al., 2009). This implies that changes in relative abundance of plasma proteins corresponding to PIVKA-II concentration may be reflecting diverse homeostatic responses to subclinical vitamin K status in a generally undernourished pediatric population.

In this study, a positive association was observed between PIVKA-II, measured by an immunoassay specific for abnormal prothrombin, and coagulation cofactor-II (or prothrombin, F2), measured by mass spectrometry. The MS-based proteomic abundance data of F2 probably reflects total prothrombin, including heterogeneous forms of under-carboxylated prothrombin, as well as completely carboxylated prothrombin. As far as we know, no comparable studies have examined the relationship between PIVKA-II and total prothrombin in healthy children.

Based on experimental or clinical studies, warfarin (vitamin K antagonist) or prenatal anticonvulsants can induce VKD, determined by an increased PIVKA-II, but only slightly decrease total prothrombin (Bach et al., 1996; Howe et al., 1999; Motohara et al., 1985). It is likely that the mild VKD in children observed in this study resulted in slightly increased abnormal forms of prothrombin rather than altering hepatic synthesis and release of normal prothrombin. This is supported by the result of proteomics data showing that MS-based total prothrombin (F2) was only 3.6% more abundant in children with VKD relative to children with vitamin K sufficiency (Table 3). Notably, none of the detected hepatic pro- and anti-coagulating VKDPs, other than F2, were associated with PIVKA-II, suggesting that suboptimal VKD status did not affect abundance of other hepatic-derived vitamin K-dependent clotting factors possibly due to their higher affinities with ϒ-glutamyl carboxylase than prothrombin (Stanley et al., 1999).

Proteins of extrahepatic origins were negatively associated with PIVKA-II. Vascular endothelial cadherin 5 (CDH5) is a calcium-dependent cell adhesion protein, controlling the vascular integrity or permeability of intercellular tight junctions in blood vessels (Dejana et al., 2009; Vestweber, 2008). Voltage-dependent calcium channel α2/δ1 (CACNA2D1) is a subunit of a transmembrane calcium channel that mediates intracellular Ca^2+^ influx and initiates diverse physiological events, including muscular contraction, excitation of neurons, and release of hormones or neurotransmitters (Hofmann et al., 1999). This subunit is expressed in excitable cells of diverse tissues including brain, heart, and skeletal muscle (Klugbauer et al., 2003).

Gelsolin (GSN) is a Ca^2+^-dependent protein that regulates actin filament assembly or disassembly in cytoplasm and efficiently severs actin present in plasma (Furukawa et al., 1997; Lind et al., 1986). A low level of GSN is associated with pathological conditions such as hemolysis, endothelial injury, apoptosis, and inflammation with poor clinical outcomes (Li et al., 2012). Mechanisms are not clear by which abundance of these calcium dependent proteins inversely associate with PIVKA-II. It can be postulated that they may involve changes in local tissue environments induced by disturbed functions of extrahepatic VKDPs. For example, some VKDPs, such as osteocalcin and matrix Gla protein (MGP), localize in bone, cartilage, vessels and smooth muscle where they regulate mineralization and calcification of extracellular matrix (Ferland, 2012a; Krueger et al., 2009; Shea and Holden, 2012; Theuwissen et al., 2012). Still, more studies are warranted to examine these relationships.

Unexpectedly, proteins primarily of red blood cell origin (hemoglobin subunits alpha1, delta, and beta) were positively associated with PIVKA-II (all q < 0.22). Hemoglobin is the most abundant protein in erythrocytes and is released into plasma when red cells undergo intravascular hemolysis (Schaer et al., 2013). Cell-free plasma hemoglobin is cytotoxic, inducing endothelial dysfunction, inflammation, and oxidative stress (Rother et al., 2005). Endogenous erythrocyte antioxidative enzymes, peroxiredoxin 2 (PRX2) and catalase (CAT), and carbonic anhydrase II (CA2), an enzyme that catabolizes carbon dioxide and adjusts blood pH, were also positively associated with PIVKA-II (Backman, 1981; Han et al., 2012; Johnson et al., 2005; Kuo et al., 2005; Lehenkari et al., 1998). Strong correlations among hemoglobin subunits and endogenous enzymes (all r > 0.7 in Fig. 1) suggest that these proteins may be closely co-regulated in a common biological process.

Limited studies have examined the link between vitamin K status and the stability of red blood cells. A study reported that long-term warfarin (vitamin K antagonist) intake induced cytokine expression in peripheral granulocytes and erythrocyte CAT activity in rats, suggesting VKD induced inflammation in immune cells and oxidative-stressed erythrocytes (Belij et al., 2012). Although we did not find evidence of systemic inflammation in our study (Table 1), moderate positive associations between PIVKA-II and PRX2, CAT and CA2 suggest that VKD may be indirectly related to oxidative stress and hemolysis in the intravascular compartment.

Risk factors of elevated PIVKA-II concentration in this study population are unclear. There were no apparent differences in intakes of dark green leafy vegetables, a major source of vitamin K, or other food groups between vitamin K sufficient and deficient children. Interestingly, children with elevated PIVKA-II showed higher concentrations of triglyceride and LDL/total cholesterol than children with a normal PIVKA-II, suggesting lipid metabolism could be associated with elevated PIVKA-II. After intestinal uptake, vitamin K1 (phylloquinone) is mainly transported by triglyceride-rich lipoproteins (TRL) to the liver and extrahepatic tissues (Sadowski et al., 1989; Shearer et al., 1974; Yan et al., 2005).

Some studies suggest that different genotypes of apolipoprotein E, a ligand for TRL or LDL receptors, is responsible for the extent of cellular uptake of lipoproteins, ultimately influencing hepatic or extrahepatic vitamin K status (Kohlmeier et al., 1995; Weintraub et al., 1987). While mechanisms remain unclear, variations in lipid/cholesterol metabolism may increase risk of subclinical VKD in children (Booth and Rajabi, 2008).

To our knowledge, this is the first study to take a systematic *-omics* approach to explore biological responses to vitamin K status in a free-living population of children. We previously reported plasma proteins associated with some micronutrients and inflammation biomarkers (Cole et al., 2013; Lee et al., 2015; Schulze et al., 2015; West et al., 2015), but distinct proteins associated with PIVKA-II from the plasma proteins of other micronutrients and health indicators suggest that plasma proteomics can identify proteins or biological processes specific to vitamin K status.

Quantitative multiplex proteomics enabled us to profile approximately 980 plasma proteins and generate hypotheses on molecular roles of vitamin K. Within a mildly elevated, narrow range of PIVKA-II, our mass spectrometric approach with large sample size was able to detect subtle, but potentially physiologically significant differences in plasma protein abundance. Among the limitations were the cross-sectional design and sole reliance on plasma PIVKA-II concentration, which is not specific to vitamin K status, and can vary with hepatic or other pathological conditions (Liebman et al., 1984). Relatively high variability in the PIVKA-II assay may lead to misclassification of children's vitamin K status and limit our ability to identify some plasma proteins differentially abundant by vitamin K status.

We were also unable to detect extrahepatic VKDPs, such as osteocalcin and MGP, which are sensitive markers of suboptimal vitamin K status (Booth et al., 2003; Schurgers et al., 2007). Based on the triage theory by McCann and Ames, extrahepatic VKDPs are more likely to be vitamin K-depleted prior to alterations in hepatic VKDPs because dysfunction in blood coagulation can be fatal (McCann and Ames, 2009). It is possible that extrahepatic VKDPs are low in abundance and were out of the detectable range of mass spectrometry.

## Conclusions

This study revealed that plasma proteomics can identify proteins revealing known and novel biological variation to suboptimal vitamin K status in healthy children. Because vitamin K deficiency likely co-exists with lipid metabolic conditions and have public health consequence, there is an increased need to assess and monitor vitamin K status in populations. Identified protein biomarkers in this study may improve understanding of potential health risks of childhood vitamin K deficiency, especially in undernourished populations.

## Supplementary Material

Supplemental data

Supplemental data

Supplemental data

## Abbreviations Used

A1BG: α-1-B glycoprotein
CA2: carbonic anhydrase 2
CACNA2D1: voltage-dependent calcium channel alpha/delta1
CAT: catalase
CDH5: vascular endothelium cadherin 5
F2: prothrombin or coagulation factor II
FDR: false discovery rate
Gla: ϒ-carboxyglutamate
Glu: glutamic acid
GSN: gelsolin
HBA1: hemoglobin subunit A1
HBD: hemoglobin delta unit
HDL: high-density lipoprotein
iTRAQ: isobaric tag for relative and absolute quantitation
LDL: low-density lipoprotein
LME: linear mixed effects models
MGP: matrix Gla protein
MS: mass spectrometry
PIVKA-II: protein induced by vitamin K absence-II
PRDX2: peroxiredoxin 2
TRL: triglyceride-rich lipoproteins
VK: vitamin K
VKD: vitamin K deficiency
VKDPs: vitamin K dependent proteins
