# Supplementary material for: Biological Systems of Vitamin K: A Plasma Nutriproteomics Study of Subclinical Vitamin K Deficiency in 500 Nepalese Children
Source: OMICS. 2016 Apr 1;20(4):214–23. doi: 10.1089/omi.2015.0178 (PMC4840917; doi:10.1089/omi.2015.0178)
Supplement: Supplemental data [file Supp_Table2.pdf]

Supplementary Data

Supplementary Table S2. Plasma Proteins Associated with Plasma PIVKA-II in Nepalese Children 6–8 Years of Age (q < 0.20)

Protein name	*Gene symbol*	n[a]	*Percent Change*[b]	*P*[c]	q[d]	$R^{2,e}$	*Accession*
Prothrombin or coagulation factor II	F2	474	137.5 (50.1, 275.8)	0.0002	0.0556	0.29	4503635
Cadherin 5, type 2 (vascular endothelium)	CDH5	474	–39.8 (–54.6, –20.1)	0.0004	0.0649	0.29	166362713
Calcium channel, voltage-dependent, alpha 2/delta subunit 1	CACNA2D1	200	–46.0 (–61.7, –23.8)	0.0005	0.0649	0.30	54112390
Gelsolin	GSN	467	–38.6 (–53.8, –18.6)	0.0007	0.0895	0.30	4504165
Hemoglobin, delta	HBD	211	11.7 (4.7, 19.1)	0.0008	0.0921	0.27	4504351
Tubulin, alpha 4a	TUBA4A	245	40.9 (14.9, 72.6)	0.0010	0.1033	0.30	17921989
Carbonic anhydrase II	CA2	419	18.6 (6.9, 31.6)	0.0012	0.1155	0.27	4557395
Multimerin 2	MMRN2	420	–33.7 (–48.4, –14.8)	0.0013	0.1155	0.27	221316695
Alpha-1-B glycoprotein	A1BG	474	101.1 (31.0, 208.6)	0.0014	0.1162	0.29	21071030
Sex hormone-binding globulin	SHBG	460	–24.6 (–37.0, –9.8)	0.0021	0.1533	0.29	7382460
Hemoglobin, alpha 1	HBA1	460	9.6 (3.4, 16.3)	0.0022	0.1533	0.27	4504347
Anthrax toxin receptor 2	ANTXR2	352	–30.3 (–44.8, –12.2)	0.0022	0.1533	0.26	50513243
Catalase	CAT	454	20.6 (6.8, 36.2)	0.0025	0.1587	0.27	4557014
Endoplasmic reticulum protein 44	ERP44	95	80.3 (22.2, 166.1)	0.0030	0.1728	0.22	52487191
Peroxiredoxin 2	PRDX2	460	14.8 (4.8, 25.8)	0.0031	0.1728	0.28	32189392
S100 calcium binding protein A4	S100A4	96	48.7 (14.2, 93.5)	0.0032	0.1728	0.30	4506765
Histidine triad nucleotide binding protein 1	HINT1	55	49.2 (14.3, 94.8)	0.0033	0.1728	0.39	4885413
Phospholipid transfer protein	PLTP	448	–35.3 (–51.8, –13.2)	0.0037	0.1802	0.29	5453914
Phosphofructokinase, liver	PFKL	374	41.4 (11.8, 78.9)	0.0038	0.1802	0.27	48762920
Cat eye syndrome chromosome region, candidate 1	CECR1	174	67.0 (17.8,136.8)	0.0040	0.1831	0.33	29029552

PIVKA-II, protein induced vitamin K absence-II.

[a]Maximum number of observation was 474 ($n = 26$ zero values were dropped due to log2 transformation of plasma PIVKA-II).

[b]Percent change in PIVKA-II concentration with doubling (100% increase) of relative abundance of protein.

[c]*P* value for hypothesis testing of a null association between PIVKA-II concentration and relative abundance of protein using a linear mixed model.

[d]Multiple hypothesis testing was corrected using false discovery rate.

[e]Variance in PIVKA-II concentration explained by protein.
