# Supplementary material for: Biological Systems of Vitamin K: A Plasma Nutriproteomics Study of Subclinical Vitamin K Deficiency in 500 Nepalese Children
Source: OMICS. 2016 Apr 1;20(4):214–23. doi: 10.1089/omi.2015.0178 (PMC4840917; doi:10.1089/omi.2015.0178)
Supplement: Supplemental data [file Supp_Table3.pdf]

Supplementary Table S3. Differentially Abundant Plasma Proteins Between Children with Vitamin K Deficiency and Children with Vitamin K Sufficiency ($q < 0.20$)[a]

Protein name	*Gene symbol*	n[b]	*Percent difference*[c]	*P*[d]	q[e]	*Accession*
Endoplasmic reticulum resident protein 44	ERP44	97	28.0 (14.2, 43.5)	2.16×10^{-5}	0.0228	52487191
Cadherin 5, type 2	CDH5	500	−6.2 (−9.0, −3.4)	2.86×10^{-5}	0.0228	166362713
Prothrombin	F2	500	3.6 (1.7, 5.5)	0.0002	0.0612	4503635
Inhibin beta E chain	INHBE	97	30.4 (13.4, 50.0)	0.0002	0.0612	13899338
Zinc finger protein 645	ZNF645	388	10.4 (4.7, 16.4)	0.0003	0.0671	22749189
Heparin cofactor II	SERPIND1	500	5.6 (2.5, 8.8)	0.0003	0.0671	73858566
Alpha 1B-glycoprotein	A1BG	500	3.6 (1.6, 5.6)	0.0004	0.0758	21071030
Haptoglobin isoform 1	HP	354	41.4 (15.2, 73.5)	0.0009	0.1443	4826762
Lymphatic vessel endothelial hyaluronic acid receptor 1	LYVE1	479	−6.8 (−10.8, −2.7)	0.0013	0.1659	40549451
Multimerin-2	MMRN2	444	−6.1 (−9.6, −2.4)	0.0014	0.1659	221316695
Vasorin	VASN	500	−4.7 (−7.5, −1.9)	0.0014	0.1659	88702793
Filamin-C isoform b	FLNC	175	56.3 (18.0, 107.2)	0.0019	0.1988	188595687

PIVKA-II, protein induced vitamin K absence-II.
[a]Vitamin K deficiency and sufficiency were defined as PIVKA-II concentration > and ≤ 2 ng/mL, respectively.
[b]Maximum number of observation was 500.
[c]Percent difference in relative abundance of protein between children with vitamin K deficiency and sufficiency.
[d]*P* value for hypothesis testing of no difference in relative protein abundance between two groups.
[e]Multiple hypothesis testing was corrected using false discovery rate.
